# Molecular Characterization and Functional Analysis of a *Schistosoma mansoni* Serine Protease Inhibitor, Smserpin-p46

**DOI:** 10.3390/microorganisms12061164

**Published:** 2024-06-07

**Authors:** Christine N. Lee, Brooke Ashlyn Hall, Leah Sanford, Adebayo J. Molehin

**Affiliations:** 1 Biomedical Sciences Program, College of Graduate Studies, Midwestern University, Glendale, AZ 85308, USA; christine.lee1@midwestern.edu; 2 Department of Microbiology and Immunology, College of Graduate Studies, Midwestern University, Glendale, AZ 85308, USA; bhall1@midwestern.edu; 3 Arizona College of Osteopathic Medicine, Midwestern University, Glendale, AZ 85308, USA; leah.sanford@midwestern.edu

**Keywords:** *Schistosoma mansoni*, serine protease inhibitors, Smserpin-p46, functional characterization, immune modulation

## Abstract

Serine protease inhibitors are a superfamily of proteins that regulate various physiological processes including fibrinolysis, inflammation and immune responses. In parasite systems, serpins are believed to play important roles in parasite colonization, inhibition of host immune serine proteases and penetration of defensive barriers. However, serpins are less well characterized in schistosomes. In this study, a *Schistosoma mansoni* serpin (Smserpin-p46) containing a 1360 base pair open reading frame, was cloned, expressed and functionally characterized. Bioinformatics analysis revealed that Smserpin-p46 contains the key residues, structural domains and motifs characteristic of inhibitory serpins. Gene expression profiling demonstrated stage-specific expression of *Smserpin-p46* with the highest expression in adult male worms. Recombinant Smserpin-p46 (rSmserpin-p46) inhibited both human neutrophil cathepsin G and elastase, key serine proteases involved in NETosis, a program for the formation of neutrophil extracellular traps. Using specific rabbit antiserum, Smserpin-p46 was detected in soluble worm antigen preparation and was localized to the adult worm tegument. Cumulatively, the expression of Smserpin-p46 on the parasite tegument and its ability to inhibit proteases involved in NETosis highlights the importance of this serpin in parasite-host interactions and encourages its further investigation as a candidate vaccine antigen for the control of schistosomiasis.

## 1. Introduction

Schistosomiasis is a neglected tropical disease that disproportionately affects people living in low- to middle-income countries. Despite decades of large-scale control efforts, current estimates indicate that over 600 million people are infected in more than 78 countries with an additional 800 million at risk of infection [1,2]. Infections with *Schistosoma mansoni* and *S. japonicum* result in hepatointestinal schistosomiasis, while urogenital schistosomiasis is caused by *S. haematobium*. While there has been some success with the World Health Organization-recommended mass drug administration of praziquantel in certain areas of mid-high disease endemicity, the problems of unabated re-infection rates, limited drug availability and emergence of drug resistance remain [3]. The development and deployment of an effective vaccine would preclude the need for frequent mass chemotherapy and ultimately lead to a significant decrease in schistosomiasis-related morbidity and mortality in endemic areas. Human population studies and experimental infection/vaccine efficacy models in rodents and nonhuman primates have provided useful insights into the immunology of schistosomiasis [1,4]. However, the molecular mechanism(s) used by schistosomes to evade or modulate host immune response and the mechanism(s) underlying vaccine-mediated resistance against schistosomiasis are yet to be fully understood. 

Serine protease inhibitors (serpins) are a superfamily of highly conserved proteins that participate in a number of fundamental physiological processes, such as blood coagulation [5,6], fibrinolysis [7], inflammation [7], signaling cascades [8,9], immune responses [10,11], tumor suppression and hormone carriage [12]. In pathogens, serpins are believed to have specifically evolved to limit or modulate their host immune responses [13]. In parasitic helminths, serpins play important roles in parasite colonization, inhibiting the effects of host serine proteases and the penetration of defensive barriers [11,14,15,16,17,18,19]. Antigen discovery using immunomics strategies has also highlighted the potential of serpins as candidate vaccine antigens against schistosomiasis. Studies by Gaze et al. in human cohorts [20] and Driguez and colleagues in rodents [21] indicate that specific host immune responses to schistosome serpins are strongly associated with immune resistance against schistosomiasis. Although our understanding of the immunopathology of schistosomiasis has significantly increased over the past few decades, much is unknown about the precise mechanism(s) employed by schistosomes to evade their host immune system or the roles played by serpins in these processes. Of the eight complete serpin sequences in the *S. mansoni* transcriptome database, only two, 46-kDa SmSPI [22] and 56-kDa Smpi56 [23], have been characterized from *S. mansoni* with potential roles in host–schistosome interactions. Based on the important physiological roles played by these proteins, other *S. mansoni* serpins are worthy of being investigated.

The aim of this present study was to obtain and characterize the full-length coding sequence of the *S. mansoni* serine protein inhibitor, termed *Smserpin*-*p46*, from the publicly available *S. mansoni* transcriptome library using bioinformatics tools. Recombinant Smserpin-p46 (rSmserpin-p46) produced was used for further functional studies and the expression of native Smserpin-p46 was determined by immunohistochemistry.

## 2. Materials and Methods

### 2.1. Parasites

*Schistosoma mansoni* life cycle stages were procured from the Schistosome Resources Center (Biomedical Research Institute, Rockville, MD, USA).

### 2.2. S. mansoni Smserpin-p46 Sequence Identification

Source sequence encoding *S. mansoni* Smserpin-p46 (GenBank: XP_018646423.1) was obtained using BLAST with the BLASTp and BLASTn algorithm against the *S. mansoni* genome database using the published *S. japonicum* serpin, SjB10 (GenBank: FN320630.1), sequence as a query. Sequence accuracy was validated by checking for the presence of start and stop codons, the consensus amino acid length for serpins (350–450) and the presence of two amino acid motifs described as highly conserved for serpins: NAVYFKG and DVNEEG. The hypothetical isoelectric point and molecular weight were identified using the ExPASy Compute pI/Mw tool.

### 2.3. Bioinformatics Analysis

The deduced amino acid sequence of the full-length *Smserpin*-p46 was submitted to BLASTp to determine the percentage homology with other known serpins. To gain probable insight into functionality, the amino acid sequence was scanned in silico against motif entries such as SignalP 5.0 Server (signal peptide) [24], SecretomeP 2.0 (ASPIRER) [25,26], TMHMM 2.0 (transmembrane domains) [27] and the Random Forests platform (N- and O-glycosylation sites) [28]. The reactive center loop was determined based on the consensus sequence 20/21 residue peptide “p17 [E]-p16 [E/K/R]-p15 [G]-p14 [T/S]-p13 [X]-p12-9 [AGS]-p8-1 [X]-p1′-4′”, the putative scissile bond, and the P1 residue was predicted based on conserved features [29,30]. The phylogenetic tree was constructed using the maximum likelihood method (500 bootstrap replications) with the MUSCLE algorithm in MEGA7 [31] to assess the evolutionary relationships among parasitic helminth serpin orthologs. Multiple sequence alignment of sequences with serpin homologs from other helminths was carried out using Clustal Omega [32]. GenBank accession numbers of all sequences used for multiple sequence alignment are presented in Table S1. The 1-, 2- and 3-dimensional structure of Smserpin-p46 was predicted using the iCn3D [33] and Phyre2 [34], and QMEANDisCO [35] was used to estimate model reliability and quality prediction. A 3-dimensional structure simulation was performed by SwissModel [36] and edited by EZMol [37]. 

### 2.4. Stage-Specific Expression of Smserpin-p46

Total RNA was extracted from *S. mansoni* life cycle stages using the RNeasy Mini kit (Qiagen, Germantown, MD, USA) and cDNA was synthesized from the total RNA using a Maxima First Strand cDNA Synthesis Kit for RT-qPCR (Thermo Fisher Scientific Inc., Waltham, MA, USA). The levels of *Smserpin*-p46 transcripts in different life cycle stages were quantified using PowerTrack^TM^ SYBR Green Master Mix for qPCR (Thermo Fisher Scientific Inc., USA) on a Bio-Rad CXF96 real-time PCR analyzer platform (Bio-Rad Laboratories, Hercules, CA, USA) using gene-specific primers. The forward and reverse primers used were 5′-GGGAAAGTCCATTTGAGCCT-3′ and 5′-CATGGCACCAGTTAGAGACCA-3′, respectively. Ubiquitin recognition factor in ER-associated degradation protein 1 (UFD1) [38] was used as a housekeeping gene with the forward primer 5′-GCGGTACAGGTTATCGGTTAG-3′ and the reverse primer 5′-ACTTCCAGGTTGATAATTGTAGTTTG-3′. The cycling conditions were 95 °C for 2 min for enzyme activation followed by 40 cycles of 95 °C for 15 s for denaturation and 60 °C for 60 s for annealing/extension. The dissociation conditions were 95 °C for 15 s, 60 °C for 1 min and 95 °C for 15 s. Melting curve analysis was performed at 65 to 95 °C. The expression of *Smserpin-p46* in each stage was normalized to the expression level of UFD1 using the formula of 2^−ΔΔCt^. Relative gene expression levels of *Smserpin-p46* were compared across all life cycle stages. All reactions were carried out in three biological replicates.

### 2.5. Construction of Recombinant Smserpin-p46

The full-length *Smserpin-p46* cDNA was amplified from adult worm cDNA using the following primers flanking the open reading frame: forward primer (5′-CCGCGGATCCGGAATGTGTATAAGATTTTCATCAAAAGTTGATG-3′) and reverse primer (5′-GCTGGCGGCCGCTTGTTATGATGATATAATTGGTTCAATAACTTTCGC-3′) which incorporated *BamHI* and *NotI* restriction sites (underlined), respectively. The amplification reaction was carried out using a thermal cycling profile of 95 °C for 30 s, 98 °C for 10 s, 30 cycles of primer-dependent annealing temperature for 30 s, 72 °C for 1 min and 72 °C for 2 min. The amplified PCR products were analyzed on a 1.2% agarose gel, gel-purified and cloned into the pGEX4T-3 prokaryotic expression vector containing an N-terminal GST tag (Millipore Sigma, St. Louis, MO, USA). 

### 2.6. Expression and Purification of Recombinant Smserpin-p46

The recombinant pGEX4T-3/Smserpin-p46 was transformed into Novagen *Escherichia coli* Rosetta 2 (DE3) chemically competent cells (MilliporeSigma, Burlington, MA, USA). The expression of the recombinant Smserpin-p46 (rSmserpin-46) was induced at 37 °C by adding isopropyl β-d-1thiogalactopyranoside (IPTG, Thermo Fisher Scientific Inc., USA) to a final concentration of 0.5 mM and then culturing the cells overnight. Cells were harvested by centrifugation at 500× *g* at 4 °C for 10 min and then resuspended in lysis buffer (1× PBS and 1% Triton X-100, pH 7.4) and sonicated. The suspension was then centrifuged at 15,000× *g* at 4 °C for 5 min and the supernatant was incubated with a Pierce^TM^ Glutathione Spin column (Thermo Fisher Scientific Inc., USA) at 4 °C for 1.5 h. After washing the column with 50 mL of wash buffer (50 mM Tris and 150 mM NaCl, pH 8.0), the recombinant protein was eluted using 5 mL of elution buffer (50 mM Tris, 150 mM NaCl and 10 mM reduced glutathione pH 8.0). The purified protein was buffer-exchanged in 1× PBS (pH 7.4), protein concentration was determined using the PIERCE™ BCA Protein Assay Kit (Thermo Fisher Scientific Inc., USA) and purity was visualized on 10% Mini-PROTEAN^TM^ TGX Stain-Free SDS-PAGE gels (Bio-Rad Laboratories, USA). To further confirm the expression and purification of the recombinant protein, Western blots were performed using a monoclonal mouse anti-GST antibody (1:275) (Santa Cruz Biotechnology, Dallas, TX, USA) as a primary antibody followed by incubation with a secondary antibody, goat anti-mouse IgG (H+L) highly cross-absorbed secondary antibody, Alexa Fluor 647 (1:2500) (Invitrogen, Waltham, MA, USA).

### 2.7. Production of Rabbit Anti-rSmserpin-p46 Polyclonal Antibody

New Zealand white rabbits were immunized three times at 3-week intervals by intramuscular injection. Prior to the first immunization, sera were collected and stored at −20 °C. Each animal was immunized with purified rSmserpin-p46 formulated in complete Freund’s adjuvant for the first injection and in incomplete Freund’s adjuvant for the remaining injections. Blood was collected 1 week after the last injection and serum was obtained and stored at −20 °C until needed. 

### 2.8. Preparations of Soluble Parasite Antigens

Crude soluble worm antigen preparation (SWAP) was carried out as previously published [39]. Briefly, freshly perfused adult worms were sonicated in cold 1 mL suspension buffer (2 mM EDTA, 1 mg/mL lysozyme, 10 μM leupeptin, 1 μM Pepstatin-a, 1 μg/mL Dnase I, 5 mM MgCl_2_ and 10 μg/mL RNase A in PBS, pH 7.4). The homogenate was then centrifuged at 15,000× *g* at 4 °C for 30 min followed by recovery of the supernatant containing soluble antigens. The protein concentration was determined using the PIERCE™ BCA Protein Assay Kit (Thermo Fisher Scientific Inc., USA).

### 2.9. Immunological Detection of Native Smserpin-p46 Protein

*S. mansoni* soluble worm antigens were electrophoresed on Mini-PROTEAN^TM^ TGX Stain-Free SDS-PAGE gels (Bio-Rad Laboratories, USA) and electrically transferred onto polyvinyl difluoride (PVDF) membranes (Bio-Rad Laboratories, USA). The membranes were blocked with 0.5× Blocker FL Fluorescent blocking buffer (Thermo Fisher Scientific, USA) for 30 min and then incubated with either anti-rSmserpin-p46 rabbit sera or pre-immune sera (1:1000) at RT for 1 h. Membranes were washed with 1× TBS (50 mM Tris, 200 mM NaCl and 0.1% Tween, pH 7.6) and incubated with goat anti-rabbit IgG (H+L) highly cross-adsorbed secondary antibody, Alexa Fluor^TM^ Plus 488 (1:2500), at RT for 1 h. Membranes were washed, air dried and scanned with the ChemiDoc XRS Imaging System to visualize the bands (Bio-Rad Laboratories, USA).

### 2.10. Immunolocalization of Smserpin-p46 by Confocal Microscopy

The expression and localization of the native Smserpin-p46 protein in *S. mansoni* adult worms was assessed by IFA using anti-rSmserpin-p46 rabbit sera as previously published [40,41] with slight modifications. Freshly perfused adult male worms were fixed in 4% paraformaldehyde in PBS (pH 7.4) for 30 min at RT and then washed 5 times with PBS. The worms were subsequently incubated twice at RT in 50 mM NH_4_Cl followed by 4 h incubation in blocking buffer (2% goat serum, 0.1% fish gelatin, 1% BSA, 0.1% Triton X-100 and 0.05% Tween-20 in PBS, pH 7.4). After blocking, the worms were washed in PBS and then incubated overnight with anti-rSmserpin-p46 rabbit sera (1:300) or pre-immune sera (1:300) at 4 °C. After five washes in PBS, preparations were incubated with mouse anti-rabbit Alexa Fluor^TM^ 488 (1:500) for 2 h at 4 °C. Alexa Fluor™ Plus 647 phalloidin (Thermo Fisher Scientific, USA) at 0.5× was added during the last 30 min of the secondary antibody incubation period. Samples were then washed thoroughly with PBS and mounted onto glass slides with ProLong™ Diamond Antifade Mountant with DAPI (Thermo Fisher Scientific, USA). Slides were examined using a Leica Stellaris 5 confocal microscope (Leica Microsystems Inc., Deerfield, IL, USA) under a 10× objective. Leica Application Suite X (Leica Microsystems Inc., USA) was used in viewing and all images were taken at room temperature.

### 2.11. Analysis of the rSmserpin-p46 Protease Inhibitory Activity

A selection of neutrophil serine proteases was used to determine the inhibitory activity and specificity of rSmserpin-p46. Human neutrophil cathepsin G and elastase inhibition assays were carried out using the SensoLyte^®^ 520 Cathepsin G Assay and SensoLyte^®^ Rh110 Elastase Assay kits (AnaSpec, Fremont, CA, USA), respectively, according to the manufacturer’s instructions. All the screening assays were performed at RT in a 100 μL reaction volume of an appropriate buffer. In brief, 0.685 µM of rGST-Sma46 was incubated with either 0.4 µg/mL of cathepsin G or 0.25 ng/mL of elastase in a 96-well plate. After adding the appropriate enzyme substrate, the proteolytic activity was measured over a 1 h period at RT as relative fluorescent units (RFUs) in a BioTek Cytation 3 spectrophotometer (US BioTek laboratories, Shoreline, WA, USA). Fluorescent intensity was measured at 490 nm excitation and 520 nm emission. All assays were carried out in triplicate and the values obtained were corrected for background fluorescence and expressed relative to the fluorescence obtained in the absence of inhibitor. The inhibitors provided in the assay kits were used for positive control wells.

### 2.12. Statistical Analysis

Statistical analyses were performed using GraphPad Prism version 9.5.1 (GraphPad, Boston, MA, USA). Data were expressed as mean ± standard error of the mean. Changes in gene expression were assessed by one-way ANOVA with post hoc Tukey testing (*p* ≤ 0.05). The inhibitory effect of rSmserpin-p46 was assessed by Student’s independent *t*-test.

## 3. Results

### 3.1. Bioinformatics Characterization of Smserpin-p46

**Figure 1:**
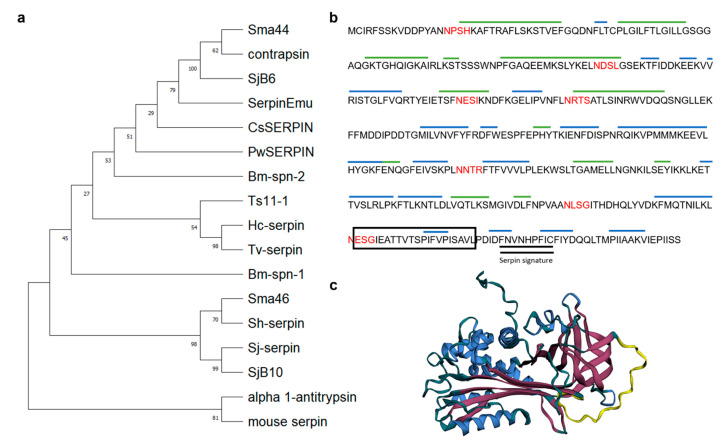
Bioinformatic analysis of Smserpin-p46. (**a**) Phylogenetic analysis of amino acid sequences for Smserpin-p46 and other parasitic helminths. The phylogenetic tree was constructed using the MUSCLE algorithm in MEGA. (**b**) Predicted amino acid sequence of Smserpin-p46 showing conserved alpha helices and beta strands. Green lines are α-helices and blue lines are β-sheets. Sequences highlighted in red are N-glycosylation sites. Sequences in the box represent the RCL while double bold lines represent the serpin signature. (**c**) The predicted three-dimensional structure of the Smserpin-p46 protein. The protein is shown in its native state with the exposed RCL (yellow) being accessible to target protease. The blue color represents the α-helices while magenta represents the β-sheets.

**Figure 2:**
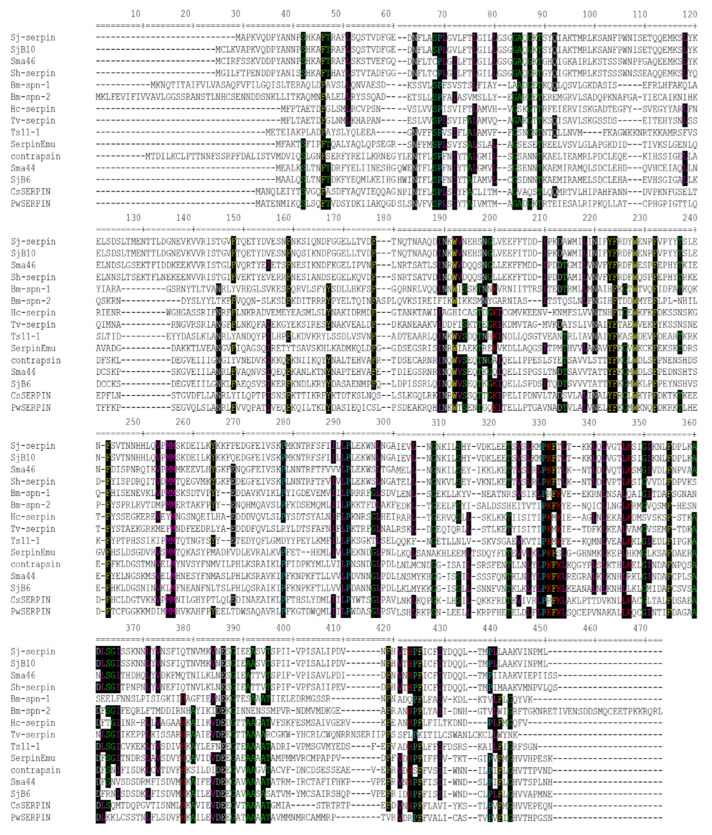
Multiple sequence alignment of Smserpin-p46 with other parasitic helminth orthologs. Sj-serpin, SjB10, SjB6 (*S. japonicum*); contrapsin (*S. mansoni*); Sma46 (*Smserpin-p46*); Sh-serpin (*S. haematobium*); Bm-spn-1, Bm-spn-2 (*Brugia malayi*); Hc-serpin (*Haemonchus contortus*); Tv-Serp (*Trichostrongylus vitrinus*); Ts11-1 (*Trichinella spiralis*); CsSERPIN (*Clonorchis sinensis*); PwSERPIN (*Paragonimus westermani*). All GenBank accession numbers are shown in Table S1.

Bioinformatics analysis showed that the *Smserpin-p46* gene sequence contains an open reading frame of 1360 base pairs encoding protein of 406 amino acid residues with predicted molecular weights of 46.01 kDa and an isoelectric point (pI) of 5.75. Predicted protein sequence analysis revealed that the sequence was complete and contained the three conserved serpin motifs, namely a hinge region (P17-P9), a signature shutter region and a reactive center loop (RCL) region (Figure 1c). The hinge region of the Smserpin-p46 RCL contained the consensus sequence typical of inhibitory serpins “P_17_ [E]-P_16_ [E/K/R]-P_15_ [G]-P_14_ [T/S]-P_13_ [X]-P_12–9_ [AGS]-P_8–1_ [X]-P_1′_–_4′_”[30], with residue variation in positions P_14_ and P_16._ The amino acids that form the active site scissile bond (P1-P1′ position) are serine and alanine. The Smserpin-p46 sequence did not contain transmembrane domains nor N-terminal signal peptides, indicating that alternative methods of secretion are used by this protein. Smserpin-p46 was found to have six potential N-glycosylation sites (16NPSH19, 97NDSL100, 134NESI137, 152NRTS155, 249NNTR252, 325NLSG328 and 349NESG352) and three O-glycosylation sites (75S, 78S and 79S) (Figure 1b). Analysis of the secondary structure showed that the Smserpin-p46 protein had 13 α-helices and 9 β-sheets. The QMEANDisCo score (measured between 0 and 1) of the predicted tertiary structure of Smserpin-p46 was 0.81 ± 0.05, demonstrating that the predicted structure is correctly modeled. Multiple sequence and tertiary structure alignment of Smserpin-p46 with other known parasitic helminth serpins showed 20–30% homology (Figure 2) with the highest sequence homology to *S. haematobium* serpin (GenBank: AAA19730). The Smserpin-p46 sequence showed lower overall sequence homology to serpins from other animals and humans. A phylogenetic tree was constructed using serpin orthologs from parasitic helminths and mammals. Analysis showed that Smserpin-p46 has the closest evolutionary relationship with *Schistosoma haematobium Sh-serpin* (Figure 1a).

### 3.2. Transcription Profiling and Production of Recombinant Smserpin-p46

**Figure 3:**
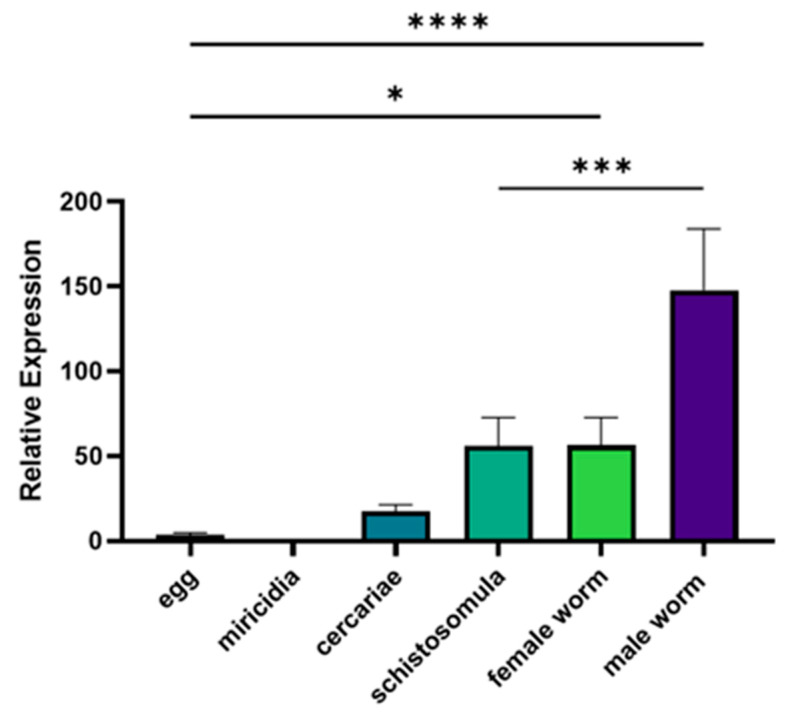
Gene expression of *Smserpin-p46* gene in *S. mansoni* life cycle stages. Transcription levels of *Smserpin-p46* were determined using SYBR real-time qPCR normalized with the expression of the *UFD1* gene in each life cycle stage. Error bars represent mean ± standard error of mean (SEM) from three biological replicates. **** *p*-value ≤ 0.0001, *** *p*-value ≤ 0.001, * *p*-value ≤ 0.05.

**Figure 4:**
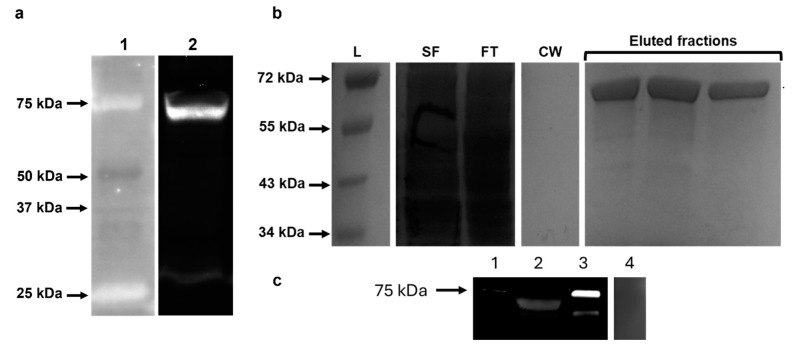
Western blot analysis of expressed recombinant GST-tagged Smserpin-p46 (rGST-Smserpin-p46) and detection of native Smserpin-p46 in parasite antigens. (**a**) rSmserpin-p46 was successfully expressed in *Escherichia coli* (lane 2). Lane 1 = Bio-Rad precision plus unstained protein ladder (**b**) Purification of rGST-Smerpin-p46 from soluble cell lysate following protein expression using Glutathione Spin column. Representative samples were analyzed by Coomassie-stained SDS-PAGE. L = NEB color protein standard, SF = filtered soluble fraction, FT = flow through, CW = crossflow waste. (**c**) Immunoblotting detecting native Smserpin-p46 in soluble worm antigen preparations (SWAP) of adult worms using anti-rSmserpin-p46 rabbit serum. Lane 1 = Bio-Rad precision plus unstained protein ladder, lane 2 = SWAP, lane 3 = rGST-Smserpin-p46, lane 4 = SWAP probed with rabbit pre-immune serum.

Stage-specific transcription of *Smserpin-p46* was assessed by real-time qPCR using cDNA reverse transcribed from RNA samples isolated from *S. mansoni* life cycle stages. Our results indicated that *Smserpin-p46* was expressed in all life stages examined (adult male, adult female, schistosomula, cercaria, miracidium and egg). The highest expression was detected in the adult male with the transcription level of the *Smserpin-p46* gene being significantly higher (*p* ≤ 0.0001) (approximately 3- to 15-fold) than all other life cycle stages’ expression (Figure 3). We successfully overexpressed GST-tagged rSmserpin-p46 in a prokaryotic expression system using IPTG. Western blot analysis using an anti-GST tag antibody showed that the GST-tagged recombinant protein was expressed at the expected molecular weight of approximately 70 kDa and purified (Figure 4a,b).

### 3.3. Detection of Native Smserpin-p46 in Soluble Worm Antigens and Tegument

**Figure 5:**
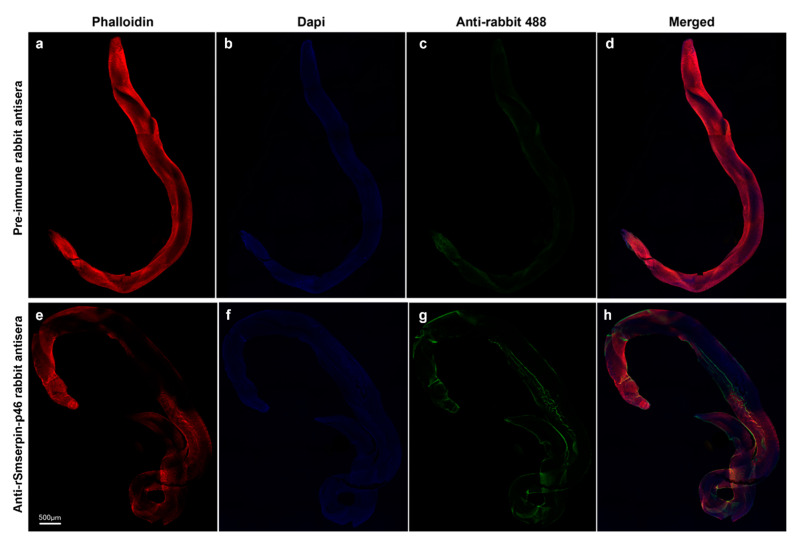
Immunolocalization of native Smserpin-p46 in *Schistosoma mansoni* adult worm. Representative stitched images of adult male worms are shown. (**a**–**d**) Fixed adult worm probed with pre-immune rabbit serum as primary antibody, (**e**–**h**) fixed adult worm probed with anti-Smserpin-p46 serum as primary antibody. The images were taken using a Leica Stellaris 5 confocal microscope under a 10× objective and analyzed with Leica Application Suite X.

Western blot analysis using antibodies raised in rabbits to the recombinantly-expressed GST-tagged Smserpin-p46 was carried out using the soluble worm antigen preparations (SWAPs) from the adult worms. Western blotting using rabbit anti-rSmserpin-p46 sera detected native Smserpin-p46 of an approximate molecular weight of 70 kDa in the parasite antigen preparations (Figure 4c). The rabbit anti-rSmserpin-p46 sera also showed strong reactivity against the recombinant protein. The rabbit anti-rSmserpin-p46 sera showed no reactivity with any band corresponding to the molecular weight of glutathione S-transferase in the SWAP. Pre-immune sera did not react with the soluble antigen worm preparation (Figure 4c). To investigate the location of Smserpin-p46 within the adult worm, we used whole-mount immunolocalization of freshly perfused worms. Using the rabbit anti-rSmserpin-p46 serum, Smserpin-p46 was localized to the tegument of the adult male worm while reaction with pre-immune sera was negative (Figure 5).

### 3.4. Recombinant Smserpin-p46 Exhibited Inhibitory Profile

**Figure 6:**
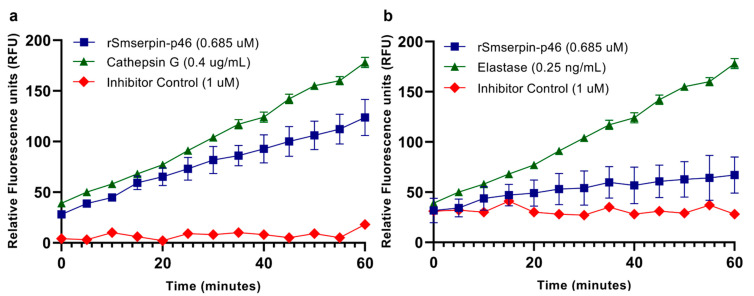
Inhibitory activity of rSmserpin-p46 against serine proteases. Inhibitory effect of rSmserpin-p46 against human (**a**) neutrophil cathepsin G and (**b**) neutrophil elastase. rSmserpin-p46 inhibited the enzymatic activities of both cathepsin G (*p* = 0.042) and elastase (*p* = 0.0002) over time (blue lines). The assays shown in green represent 100% enzyme activity against which all assays were compared. Specific inhibitor controls are shown in red lines. All values were corrected for background fluorescence and assays were performed in triplicate. Error bars represent the mean ± standard error of mean.

Neutrophils use NETosis to immobilize and kill invading helminth larvae, and larvae that successfully evade neutrophil extracellular traps (NETs) will migrate to the lungs for further development [42,43]. Using two key serine proteases involved in NETosis, we showed that r-Smserpin-p46 is a functional inhibitor with distinct specificities. Our result showed that Smserpin-p46 significantly inhibited the activity of both human neutrophil cathepsin G (*p* = 0.042) and elastase, with the higher inhibition recorded against elastase (*p* = 0.0002) (Figure 6).

## 4. Discussion

Inhibitory serpins are suicide substrate high-molecular-weight protein inhibitors of serine proteases [13,29]. The reactive center loop (RCL) contains the recognition sequence for the active site of the target protease. The mechanism of inhibition involves the kinetic trapping of an enzyme intermediate leading to significant irreversible conformational change. Serpins have been identified and characterized in many parasite systems including helminths and they have been shown to play important roles in host immune modulation and/or evasion [11,13,44,45]. 

In this study, we described the molecular features and functions of Smserpin-p46 from *Schistosoma mansoni*. Sequence analysis showed that the *Smserpin-p46* gene contained the conserved functional features and domains that are characteristic of inhibitory serpins, such as serpin motif, serpin signature and the RCL [13,29]. Furthermore, Smserpin-p46 contained 406 amino acid residues with a predicted molecular weight of 46 kDa and a native molecular weight of 70 kDa, consistent with other members of the serpin superfamily [30]. Further bioinformatics analysis demonstrated that Smserpin-p46 lacked an N-terminal signal peptide, C-terminal extensions and transmembrane domain. This suggests that the Smserpin-p46 protein might be cytosolic or secreted through the non-classical secretory pathway as demonstrated with some other serpins [46,47]. Additionally, the predicted secondary and tertiary structure of Smserpin-p46 showed that the polypeptide contained thirteen α-helices and nine β-sheets with an exposed RCL, thereby making it accessible to target proteases [48]. These observations support the argument that Smserpin-p46 belongs to the inhibitory class of serpins.

Serpin inhibition mechanisms depend on an essential residue (P1) and structure (hinge region, residues P15-P9) within the RCL. The composition of the amino acid residues within the RCL active site determines whether the serpin is inhibitory or non-inhibitory as well as its specificity if inhibitory [29,30]. Based on the consensus 20/21 residue peptide of the RCL, inhibitory serpins usually contain glycine at position 15 (P15) while P14 is usually a serine or threonine. Positions 12-9 are known to be short side-chain residues. These key residues allow for the efficient insertion of RCL into the A β-sheet, a critical step in protease inhibition [29,30]. The corresponding region in non-inhibitory serpins deviates from this consensus. Analysis of Smserpin-p46 RCL showed that it contained residues consistent with inhibitory serpins. Further analysis of the highly conserved hinge region of Smserpin-p46 showed that it is composed mainly of hydrophobic residues, which are believed to contribute to the necessary structural conformation required for the inhibitory activity of serpins [49]. Mutations in the hinge region abrogate the inhibitory activity of serpins [50]. Phylogenetic analysis and multiple sequence and structure alignment with orthologs from other known parasitic helminths showed that Smserpin-p46 is more closely related to a *S. haematobium* serpin (Sh-serpin) [51] which may indicate similar biochemical and biological roles between these two serpins. It is noteworthy that Smserpin-p46 is distantly related to the mammalian serpins investigated.

Full-length GST-tagged Smserpin-p46 was successfully expressed as a soluble protein in a prokaryotic expression system with a molecular size of approximately 70 kDa and was detected by Western blotting using an anti-GST specific antibody. Immunization of rabbits with purified rSmsrpin-p46 showed that it was highly immunogenic, thereby generating anti-rSmserpin-p46 serum. Native protein was detected in the soluble worm antigen preparations using rabbit anti-Smserpin-p46 serum as a single band of approximately 70 kDa, larger than the recombinantly produced protein of 46 kDa. This is not surprising as the analysis of the Smserpin-p46 sequence showed that it contains multiple N- and O-glycosylation sites indicating that the protein undergoes post-translational modifications as is the case with many other serpins [19,44,46,48]. Post-translational modifications are critical to serpin functionality and specificity [52,53].

Gene expression profiling through RT-qPCR indicated that *Smserpin-p46* was expressed in all *S. mansoni* life cycle stages examined with the highest expression in adult male worms. The stage-specific (intra-mammalian) expression of Smserpin-p46 provided some insight into its possible biological function when the parasite is inside the definitive host. Our findings from the protease inhibition assay lend support to this argument in that rSmserpin-p46 inhibited the activities of multiple human serine proteases. It is noteworthy that Smserpin-p46 inhibited the activity of lysosomal serine proteases, such as human neutrophil cathepsin G and elastase, which are predominantly expressed in neutrophil and macrophages and play important roles in inflammation and monocyte recruitment [54,55]. One of the key effector mechanisms employed by neutrophils to combat pathogens is the formation of neutrophil extracellular traps (NETs) [56] in a process called NETosis. NETs are complex webs of extracellular DNA studded with histones and cytoplasmic granular proteins such as cathepsin G and elastase [56,57]. In parasitic metazoan infections, NETosis is associated with parasite trapping, inhibition of larval development and direct parasite killing (larvicidal) [43]. Both adult and larval forms of schistosomes have been shown to be sensitive to both pancreatic and neutrophil elastase [46,58,59] while other studies have suggested that the regulation of host serine proteases by a specific parasite serpin promotes parasite invasion and survival [11,44,46,60]. We therefore propose that *S. mansoni* utilizes Smserpin-p46, at least in part, to counteract the deleterious effects of these host proteases, thereby promoting their survival within their hosts. This hypothesis is supported by the result from the IFA using anti-Smserpin-p46 serum which indicated that Smserpin-p46 is localized to the surface tegument of adult male worms of *S. mansoni*. 

In summary, we characterized some of the molecular and biochemical properties of Smserpin-p46, an inhibitory serpin form *Schistosoma mansoni.* Stage-specific *Smserpin-p46* gene expression, its localization to the parasite tegument and its inhibitory activity against human lysosomal serine proteases highlight its possible role in parasite survival, host–parasite interaction and its potential as a target for drug and/or vaccine development. High-throughput screening for specific inhibitors against rSmserpin-p46 and vaccine testing are further studies that could provide more insight into the biology of this serpin.

## Data Availability

All data generated or analyzed during this study are included in this published article.

## Supplementary Materials

The following supporting information can be downloaded at https://www.mdpi.com/article/10.3390/microorganisms12061164/s1, Table S1: GenBank Accession numbers for sequences used for multiple sequence alignment.
