# Supplementary material for: Molecular Characterization and Functional Analysis of a Schistosoma mansoni Serine Protease Inhibitor, Smserpin-p46
Source: Microorganisms. 2024 Jun 7;12(6):1164. doi: 10.3390/microorganisms12061164 (PMC11205507; doi:10.3390/microorganisms12061164)
Supplement: Supplementary file 1 [file microorganisms-12-01164-s001.zip › microorganisms-3029340-supplementary.pdf]

Supplementary Materials

Table S1: GenBank Accession numbers for sequences used for multiple sequence alignment

Serpin name	GenBank accession number	Source
A1-antitrypsin	AY256958	*Homo sapiens*
CsSERPIN	EF550965	*Clornochis sinesis*
SerpinEmu	CAD12372.2	*Echinococcus multilocularis*
Hc-serpin	ACP43576	*Haemonchus contortus*
PwSERPIN	EU014295	*Paragonimus westermani*
Sh serpin	AAA19730	*Schistosoma haematobium*
Contrapsin	CCD60352.1	*Sschistosoma mansoni*
Ts11-1	DQ864973	*Trichinella spiralis*
Tv Serp	Y12233	*Trichostrongylus vitrinus*
Bm-spn-1	U04206	*Brugia malayi*
Bm-spn-2	AF009825	*Brugia malayi*
SjB6	CAX69453.1	*Schistosoma japonicum*
SjB10	FN320630.1	*Schistosoma japonicum*
Mouse	AAH62169.1	*Mus musculus*
